# Development of Spectrofluorimetric and HPLC Methods for *In vitro* Analysis of Repaglinide

**DOI:** 10.4103/0250-474X.65029

**Published:** 2010

**Authors:** N. Kaushal, S. Jain, A. K. Tiwary

**Affiliations:** Department of Pharmaceutical Sciences and Drug Research, Punjabi University, Patiala-147 002, India

**Keywords:** Repaglinide, spectrofluorimetric method, HPLC method, analytical method validation

## Abstract

Spectrofluorimetric and high-performance liquid chromatography methods for estimation of repaglinide were developed. These methods were validated for estimation of repaglinide in tablets as well as in receptor fluid obtained during *in vitro* permeation studies. Repaglinide was observed to exhibit emission and excitation wavelengths, respectively, at 379 nm and 282 nm with linearity in the concentration range of 5-80 µg/ml. High-performance liquid chromatography analysis of repaglinide yielded retention time of 6.14 min with linearity ranging from 0.1-1.2 µg/ml concentration. Spectrofluorimetric analysis of repaglinide in tablets yielded results comparable to high performance liquid chromatography.

Repaglinide (RGE), is an oral antidiabetic drug. Tablets containing 0.5, 1 and 2 mg of RGE are available for oral administration. The methods investigated for analysis of RGE include HPLC method with UV detector[1,2] or electrochemical method by using carbon paste electrode and glassy carbon electrode[3]. Liquid chromatography-tandem mass spectrometry (LC/MS/MS) and normal phase chiral HPLC methods[4] for determination of RGE are also reported. The HPLC method reported by USP claims to be sensitive enough for the analysis of RGE. However, these procedures are expensive and inconvenient for routine analysis of RGE. Therefore, it was felt essential to develop a facile method suitable for routine analysis of RGE during early development phase of tablets/transdermal delivery systems. Further, the results of fluorimetric analysis were compared with those obtained by HPLC analysis.

Spectrofluorimeter SL-174 (ELICO, India Ltd.) was used for the fluorimetric analysis. Waters HPLC system equipped with 515 binary pump, degasser and a 2487 dual wavelength ultraviolet detector (UV), rheodyne manual injector and RP C-18 column (250×4.6 mm, particle size 5 µm) were used for HPLC analysis. The output signal was monitored and processed using Empower-2 software.

Repaglinide (RGE) (assigned purity, 99.8%) was a gift sample from M/S Torrent Pharmaceutical Ltd., Ahmedabad, India. Commercially available RGE (0.5 mg) tablets (Repalin, Cipla Pharmaceuticals Ltd., Mumbai) were procured from the local pharmacy. All the reagents used in this study were of HPLC grade and procured from Rankem, New Delhi. The water used in the mobile phase was obtained using the Millipore synergy system.

All fluorescence measurements were made using excitation and emission window of 20 nm at a sensitivity of 450 nm. The fluorescence spectra were recorded at a scan rate of 240 nm/min. Stock solution (1000 µg/ml) of RGE was prepared in methanol by sonication. Further dilutions were made using methanol and the solutions (5-100 µg/ml) were scanned for the determination of the excitation and emission maxima. The influence of pH on fluorimetric attributes of RGE was assessed by preparing solutions containing 50 µg/ml of RGE in phosphate buffer (PB) in the range of pH 1.2-12.2.

The suitability of fluorimetric analysis for estimating RGE during *in vitro* permeation experiments across rat skin was assessed by preparing its stock solution (1000 µg/ml) in methanol. These solutions were adjusted to volume with phosphate buffer (PB) pH 7.4, PB containing polyethylene glycol 400 (10% v/v) PEG and sodium azide (0.05% w/v) or PB (pH 7.4) containing constituents leached from excised rat epidermis. All solutions were scanned for excitation and emission maxima.

Mobile phase (acetonitrile:ammonium acetate, pH 4.5, 70:30 v/v) for HPLC analysis was filtered before use through a 0.22 µm membrane filter, degassed with a helium spurge for 15 min and pumped to the column maintained at 30° at a flow rate of 1.0 ml/min. The eluents were monitored at 240 nm and the data acquired was stored and analyzed using Empower-2 software.

A stock solution of RGE (1000 µg/ml) was prepared in acetonitrile (ACN). Working standard solutions of RGE (0.1-1.2 µg/ml) were prepared by suitable dilution of the stock solution with the mobile phase. Each drug solution (20 µl) was injected for HPLC determination of peak area and retention time.

Eight tablets were weighed[5] to obtain the average tablet weight and were then powdered. A sample of the powdered tablets was dissolved in methanol and diluted with methanol:phosphate buffer pH 4.0 (7:3) to obtain a solution having a concentration of 80 µg/ml. The solution was filtered through a 0.22 µm membrane filter and then injected for HPLC analysis. The same samples were subjected to spectrofluorimetric analysis.

The spectrum of a solution of RGE in methanol exhibited excitation and emission at, respectively, 282 and 379 nm (fig. 1a). The same wavelengths were found to characterize the excitation and emission maxima of RGE solutions prepared in PB in the pH range of 6.8-12.2 (figs. 1b‐e). However, the excitation and emission wavelengths, respectively, shifted to lower wavelength of 240 nm and 358 nm when solution was prepared in pH 1.2, 3.2 or 4.2 (fig. 1f, g and h). The effects of solution acidity and basicity on luminescence spectra result from the dissociation of acidic functional groups or protonation of basic functional groups associated with the aromatic portions of fluorescing molecules. Protonation and dissociation alter the relative separation of ground and excited states of the reacting molecules and thereby cause shifting of the luminescence spectra. This shift tends to be greater in fluorescence spectra than in phosphorescence spectra and are attributable to the electrostatic stabilization or destabilization of excited state, relative to the ground state. The protonation of electron withdrawing groups such as carboxy, carbonyl and pyridine nitrogen cause shifting of the luminescence spectra to longer wavelength. The electron withdrawing groups carboxy, carbonyl as well as nitrogen attached to aromatic ring of RGE seem to be responsible for shifting the emission maxima from 358 nm to 379 nm (figs. 1b‐h) when RGE was dissolved in buffers with pH increasing from 1.2-12.2. Therefore, alteration of the fluorescence spectrum due to the phenomenon of protonation or dissociation occurring during acid-base reactions of fluorescent molecules at different pH values must be considered while developing fluorimetric analysis method of RGE.

**Fig. 1:**
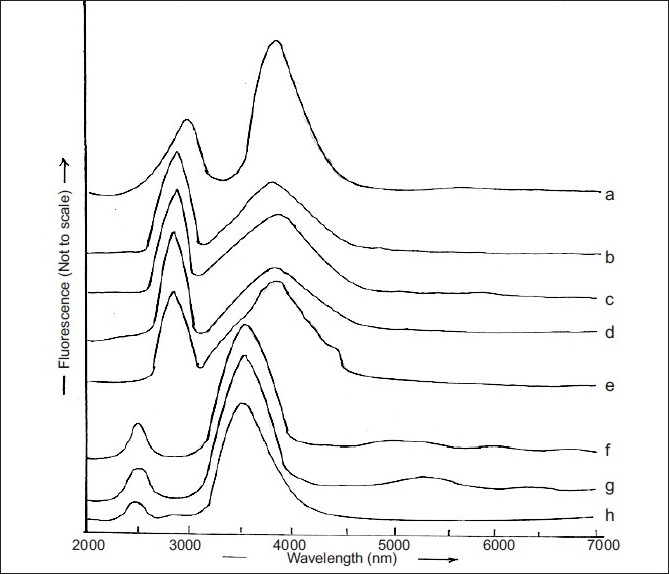
Excitation and emission maxima of RGE in different pH media. RGE solutions were prepared in methanol (a); PB pH 6.8 (b), PB pH 7.4 (c), PB pH 9.2 (d), PB pH 12.2 (e), PB pH 1.2 (f), PB pH 3.2 (g) or PB pH 4.2 (h)

RGE is a suitable candidate for formulation into transdermal drug delivery systems due to its short biological half life of 1 h and the need for maintaining constant plasma concentration over extended duration for effective control of blood glucose level. Hence, it is essential to examine the interference of skin components that often get extracted into the receptor fluid (PB, pH 7.4) for correct estimation of the amount of RGE permeated across skin during *in vitro* experiments. The fluorimetric spectrum of PB (pH 7.4) is depicted in fig. 2a. Similar spectrum was observed when PB (pH 7.4) obtained after stabilization of rat epidermis was subjected to fluorimetric analysis (fig. 2b) and PB pH 7.4 containing PEG 400 (10% v/v) and sodium azide (0.5% w/v) (fig. 2c). In addition, the fluorimetric analysis of PB (pH 7.4) obtained after 4 h of rat epidermis stabilization containing PEG 400 (10% v/v) as solublizier and sodium azide (0.05% w/v) as preservative was observed to be similar to these spectra (fig. 2d).

**Fig. 2:**
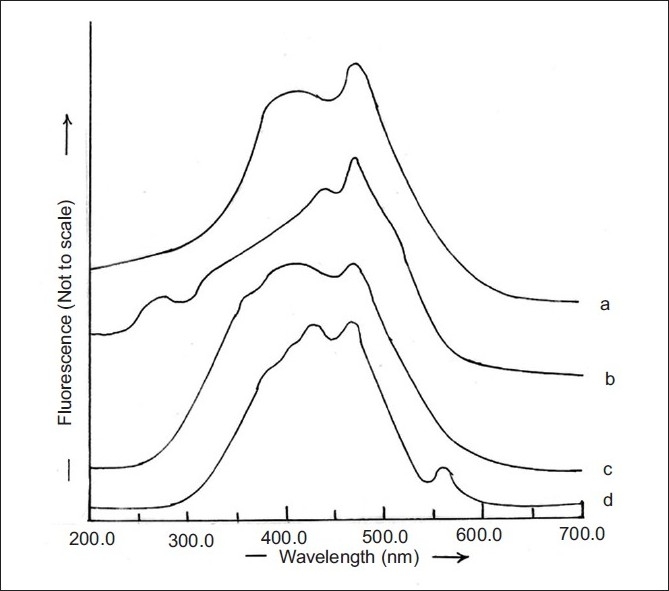
Fluorimetric spectra of PB containing different constituents. Fluorimetric spectra of PB pH 7.4 (a); PB pH 7.4 obtained after 4 h of rat epidermis stabilization (b); PB pH 7.4 containing PEG 400 (10% v/v) and sodium azide (0.5% w/v) (c) or PB pH 7.4 obtained after 4 h of rat epidermis stabilization containing PEG 400 (10% v/v) and sodium azide (0.5% w/v) (d)

The fluorimetric analysis of RGE solution (80 µg/ml) prepared in PB (pH 7.4) obtained after 4 h of stabilization of rat epidermis containing PEG 400 (10% v/v) and sodium azide (0.05% w/v) revealed excitation and emission maxima at, respectively, 282 and 379 nm (fig. 3c), which were similar to those obtained in a solution of RGE in methanol. Therefore, the fluorimetric analysis of RGE was performed using 282 and 379 nm as excitation and emission wavelength, respectively.

**Fig. 3:**
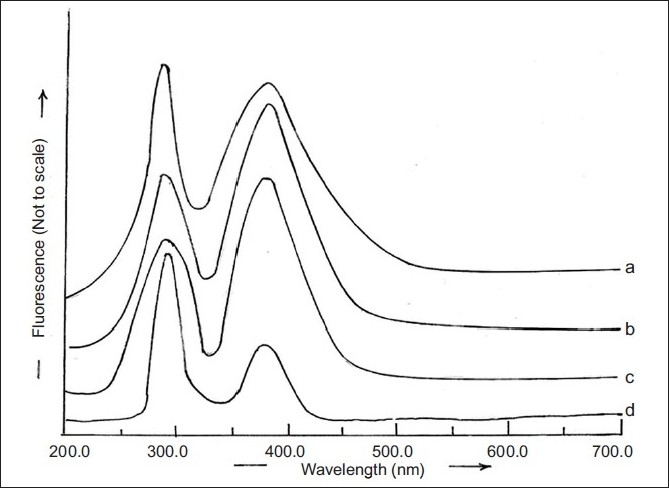
Fluorimetric spectra of different concentrations of RGE. Solutions containing 10 μg/ml (a), 40 μg/ml (b) or 80 μg/ml (c) concentration of RGE in PB (pH 7.4) obtained after 4 h of rat epidermis stabilization containing PEG 400 (10% v/v) and sodium azide (0.5% w/v) or solution of tablet containing 80 μg/ml RGE concentration (d)

The excitation and emission spectra, respectively, were observed at 282 and 379 nm after extraction of RGE from commercially available tablets. These wavelengths were similar to those observed for different concentrations of RGE in PB, pH 7.4 (fig. 3). Hence, the fluorimetric method for analyzing RGE from commercial tablets can be suggested to be specific for RGE. A standard plot of RGE was prepared in PB (pH 7.4). The plot was linear in the range of 5-80 µg/ml and was represented by the equation Y=1.9247x+17.351 (r^2^ = 0.9989).

LOD was calculated by using the formula DL=3.3 σ/S, where DL is the detection limit, σ is the standard deviation of intercepts of the regressed lines and S is the average slope of the regressed line. LOQ was calculated by the formula DQ=10 σ/S, where DQ is the limit of quantification. They were calculated to be, respectively, 6.49 µg/ml and 14.69 µg/ml. However, experimentally they were found to be, respectively, 5 µg/ml and 10 µg/ml. The inter-day and intra-day precisions for lowest middle and highest concentrations suggest that the developed fluorimetric method was precise, accurate and reproducible for analyzing RGE (Table 1).

**TABLE 1:** INTER- AND INTRA-DAY VARIATION OBSERVED DURING ANALYSIS OF REPAGLINIDE BY SPECTROFLUORIMETRIC AND HPLC METHODS

Conc (μg/ml)	Inter-day variation				Intra-day variation			
	Mean	RSD%	COV %	Accuracy %	Mean	RSD%	COV%	Accuracy%	
				Spectrofl uorimetric analysis					
10	9.987	0.056	6.26	95.34	9.965	0.091	9.12	93.89
40	39.19	0.016	1.75	99.98	38.84	0.015	5.89	101.24
80	78.88	0.020	2.04	103.78	80.03	0.087	8.75	100.76
				HPLC analysis					
0.1	0.199	0.034	7.25	102.11	0.187	0.089	8.54	102.09
0.6	0.624	0.087	2.56	99.87	0.578	0.076	5.65	97.89
1.2	1.211	0.061	7.98	104.78	1.198	0.043	12.67	99.19

RSD-Relative standard deviation; COV-Coefficient of variance. Assay Tablet a) Spectrofluorimetric method: 69.43±0.045; b) HPLC method: 71.82±0.089. Inter-day variation was assessed by analyzing lower (10 g/ml), medium (40 μg/ml) and highest (80 μg/ml) concentrations of RGE for spectrofl uorimetric method or 0.1 μg/ ml, 0.6 μg/ml and 1.2 μg/ml concentrations of RGE on three different days for HPLC method. The same concentrations in triplicate were analyzed on the first day for assessing Intra-day variation.

The retention time of 6.14 min at 240 nm (fig. 4) for RGE is in close agreement with 6.2 min reported by Ruzilawati *et al*[6] suggesting the specificity of the HPLC method. The standard plot of RGE prepared by HPLC was linear in the range of 0.1 µg/ml -1.2 µg/ml and it was represented by equation Y=18691x+232290 (r^2^ =0.9986). The LOD and LOQ, respectively, were observed to be 0.1 µg/ml and 0.4 µg/ml.

**Fig. 4:**
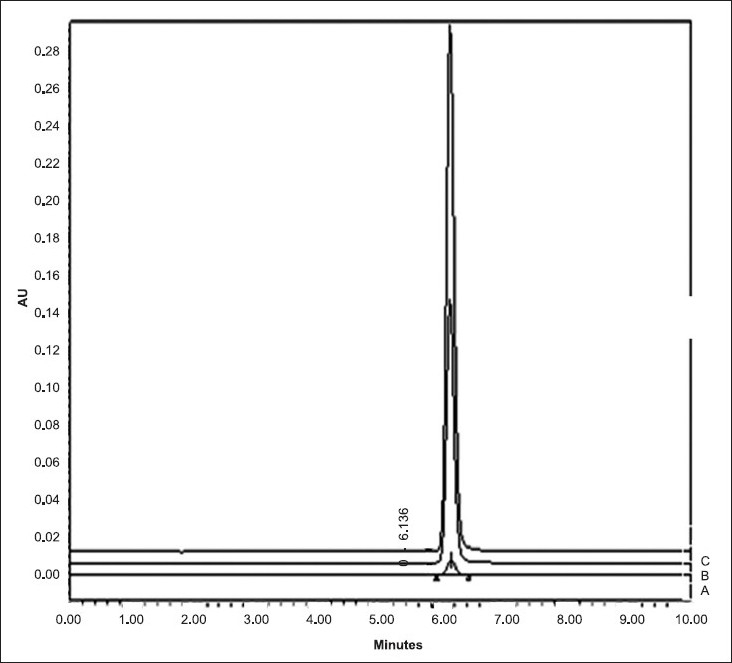
Chromatogram of RGE obtained after HPLC analysis Solutions containing 0.1 μg/ml (A), 0.6 μg/ml (B) and 1.2 μg/ml (C) concentrations of RGE in mobile phase (ammonium acetate: acetonitrile, 30:70)

The comparison of the parameters obtained after validation of the analytical methods indicated that HPLC method was more sensitive than the fluorimetric method for analyzing pure sample of RGE. However, the content of RGE in a sample obtained after extraction of RGE from commercial tablets (USP, 2007) estimated by fluorimetric analysis was 69.43±0.045 (mean±SD, n=3) and that by HPLC analysis was 71.82±0.089 (mean±SD, n=3).

Hence, although, the HPLC method exhibited better sensitivity as compared to spectrofluorimetric method, the later was comparable to the former for estimating RGE in tablets and during *in vitro* permeation experiments involving testing of transdermal dosage forms. The developed fluorimetric method could be used as facile method for *in vitro* analysis of RGE.
